# TNFα is a key trigger of inflammation in diet-induced non-obese MASLD in mice

**DOI:** 10.1016/j.redox.2023.102870

**Published:** 2023-09-01

**Authors:** Katharina Burger, Finn Jung, Anja Baumann, Annette Brandt, Raphaela Staltner, Victor Sánchez, Ina Bergheim

**Affiliations:** Department of Nutritional Sciences, Molecular Nutritional Science, University of Vienna, Vienna, Austria

**Keywords:** Fatty liver, MASH, Endotoxin, Insulin resistance, Intestinal barrier

## Abstract

Tumor necrosis factor alpha (TNFα) is thought to be a critical factor in the development of metabolic dysfunction-associated steatotic liver disease (MASLD). Here, we determined the effects of a treatment with the *anti*-TNFα antibody infliximab and a genetic deletion of TNFα, respectively, in the development of non-obese diet-induced early metabolic dysfunction-associated steatohepatitis (MASH) in mice. The treatment with infliximab improved markers of liver damage in mice with pre-existing early MASH. In TNFα^−/−^ mice, the development of early signs of MASH and insulin resistance was significantly attenuated compared to wild-type animals. While mRNA expression of proinflammatory cytokines like *interleukin 1β* (*Il1b*) and *interleukin 6* (*Il6*) were significantly lower in livers of MASH-diet-fed TNFα^−/−^ mice compared to wild-type mice with early MASH, markers of intestinal barrier function were similarly impaired in both MASH-diet-fed groups compared to controls. Our data suggest that TNFα is a key regulator of hepatic inflammation and insulin resistance associated with the development of early non-obese MASH.

## Highlights

• TNFα is a key regulator of hepatic inflammation and insulin resistance.
• A genetic deletion of TNFα attenuates the development of early signs of MASH and insulin resistance.
• A treatment with infliximab improved markers of liver damage in mice with pre-existing early MASH.

## Graphical abstract

**Figure:**
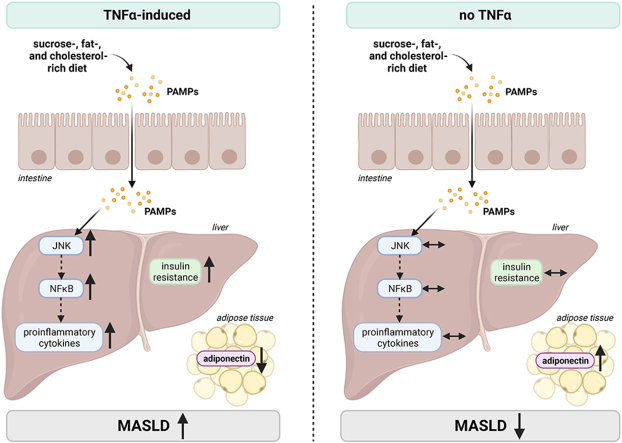

## List of abbreviations:

aSma: alpha smooth muscle actin
Adipor2: adiponectin receptor 2
ALT: alanine aminotransferase
AUC: area under the curve
Col1a1: collagen type I alpha1
C: control diet
FBS: fetal bovine serum
GTT: glucose tolerance test
IBD: inflammatory bowel disease
Il: interleukin
Irs: insulin receptor substrate
Ir: insulin receptor
JNK: c-Jun N-terminal kinase
KRH buffer: 1× Krebs-Henseleit-bicarbonate buffer supplemented with 0.2% bovine serum albumin
LPS: lipopolysaccharide
MASH: metabolic dysfunction-associated steatohepatitis
MASLD: metabolic dysfunction-associated steatotic liver disease
Mcp1: monocyte chemoattractant protein-1
MPO: myeloperoxidase
NAS: NAFLD activity score
NO: nitric oxide
NO_2_^-^: nitrite
PAI-1: plasminogen activator inhibitor 1
PCCs: peritoneal cavity cells
SEM: standard error of the means
SFC: sucrose-, fat-, and cholesterol-rich diet
Suc: sucrose
TLRs: Toll-like receptors
TNFα: tumor necrosis factor alpha
TNFR1: TNFα receptor 1
TNFR2: TNFα receptor 2
ZO-1: zonula occludens 1

## 1 Introduction

Non-alcoholic fatty liver disease (NAFLD), now referred to as metabolic dysfunction-associated steatotic liver disease (MASLD) [1], has emerged to be the most frequent liver disease in the world [2]. Indeed, it is estimated that in Europe ∼30% of the general population is affected by MASLD by now [2,3]. General overnutrition along with low physical activity and overweight have been shown to be critical in the development of MASLD and insulin resistance, the latter being one of the key risk factors for the development of MASLD [4,5]. However, studies have also shown that the number of non-obese and lean individuals, respectively, suffering from MASLD has steadily increased during the last decade [6]. Results of clinical and animal studies further suggest, that the dietary pattern and herein especially a diet rich in saturated fats and sugar as well as cholesterol may lead to the development of MASLD even in the absence of obesity [[7], [8], [9]]. Moreover, the development of MASLD has also been shown to be related to changes of intestinal microbiota composition, impairments of intestinal barrier function and elevated bacterial (endo)toxin levels in peripheral blood, the latter being associated with an induction of Toll-like receptors (TLRs) in peripheral mononuclear cells and liver tissue [10,11]. And while the understanding of the molecular mechanisms underlying the development of MASLD has markedly progressed in recent years, life-style interventions focusing on weight reduction and increased physical activity are still the main therapy of choice for most MASLD patients [12].

Already more than 20 years ago an induction and mis-regulation of the expression of tumor necrosis factor alpha (TNFα) has been linked to the development of MASLD [13] and the role of TNFα in the onset and progression has since been investigated in many studies (for overview see [14]). For instance, results of clinical studies have shown that as MASLD progresses from simple steatosis to MASH, TNFα levels increase along in blood, liver, and adipose tissue [15,16]. Furthermore, it has been shown by us and others that a genetic deletion of the TNFα receptor 1 (TNFR1) or TNFR1 and 2, respectively, significantly diminished the development of diet-induced MASLD in mice [17,18]. In ob/ob mice, the injection of *anti*-TNFα antibodies improved steatosis and inflammation as well as activity of c-Jun N-terminal kinase (JNK), shown to be critical in promoting insulin resistance, was dampened [19]. Results alike were also shown when infliximab, an *anti*-TNFα antibody, was employed in MASH rat models [20]. However, despite having by now been acknowledged as one of the causal factors that markedly contributes to the progression of MASLD and especially that of MASH [14] as well as in light of promising pre-clinical studies employing *anti*-TNFα antibodies, there are still several gaps in the understanding how TNFα adds to the onset and progression of MASLD.

Starting from this background the aim of the present study was to assess the effect of the *anti*-TNFα antibody infliximab and a genetic deletion of TNFα in a dietary mouse model of non-obese MASLD. Moreover*, ex vivo* and cell culture experiments were employed to further delineate molecular mechanisms underlying the effects of the loss of TNFα.

## 2 Methods

### 2.1 Animals and treatment

C57BL/6J and TNFα^−/−^ (B6·129S-Tnftm1Gkl/J) mice were obtained from Jackson Laboratory (Bar Harbor, ME, USA) and Janvier (Janvier SAS, Le-Genest-Saint-Isle, France), respectively. Mice were bred in a specific-pathogen-free barrier facility accredited by the Association for Assessment and Accreditation of Laboratory Animal care. All experiments were carried out under controlled conditions (12h/12h light/dark cycle, ∼24 °C, ∼55% relative humidity) with mice having free access to tap water at all times. All procedures were approved and registered by the local Institutional for Animal Care and Use Committee (“Bundesministerium für Wissenschaft, Forschung und Wirtschaft, Referat für Tierversuchswesen und Gentechnik”, Vienna, Austria). **Intervention trial 1:** After being adapted to the liquid control diet, six to eight weeks old male C57BL/6J mice were assigned to the following groups: control diet (C; 69 E% carbohydrates, 12 E% fat, 19 E% protein, Ssniff, Soest, Germany) and liquid sucrose-, fat-, and cholesterol-rich diet (SFC; 60 E% carbohydrates, 25 E% fat derived from butterfat, 15 E% protein with 50% (wt/wt) sucrose and 0.16% (wt/wt) cholesterol, Ssniff). Mice were pair-fed the respective diets for 7 weeks as detailed previously [21] and were then randomly assigned to either be treated 3 times/week i.p. with 10 mg/kg bw infliximab (*anti*-TNFα antibody (Sigma-Aldrich, Steinheim, Germany)) or vehicle (0.9% NaCl) for 1 week (n = 6–8/group). **Intervention trial 2:** Six to eight weeks old male wild-type and TNFα^−/−^ mice were randomly assigned to the following groups (n = 6–8 mice/group): wild type and TNFα^−/−^ mice, respectively, being fed the liquid C or wild type mice and TNFα^−/−^ mice, respectively, being fed the liquid SFC. After an adaptation phase to the liquid control diet, mice were pair-fed the respective diets for 9 weeks as described in detail previously [21]. In week 8, a glucose tolerance test (GTT) was performed. Mice were fasted for 6 h and after assessing fasting blood glucose concentrations, a glucose solution (2 mg/kg bw) was injected i.p. Blood was collected from the tail vein to assess blood glucose concentrations with a standard glucometer (Contour, Bayer Vital GmbH, Leverkusen, Germany). Intestinal permeability in small intestine was assessed *ex vivo* in everted small intestinal tissue sacs as described before [21]. Experimental set-ups of trials 1 and 2 are summarized in Figs. S1A and B. At the end of both interventions, mice were anesthetized with a ketamine/xylazine mixture (i.p. injection, 100 mg ketamine/kg bw; 16 mg xylazine/kg bw) and killed by cervical dislocation. Blood was collected from portal vein. Hepatic and intestinal tissue samples were collected and fixed in neutral-buffered formalin or immediately snap-frozen and stored in a −80 °C freezer.

### 2.2 Isolation of peritoneal cavity cells (PCCs) and cell culture experiments

For *ex vivo* cell stimulation experiments, PCCs being a mix of macrophages, T- and B-cells [22], were isolated from naïve 4–7 months old female C57BL/6J and TNFα^−/−^ mice (n = 4–5/mouse strain) using a protocol published previously by Ray et al. [22]. In brief, mice were killed by cervical dislocation and 5 ml of ice-cold PBS supplemented with 3% fetal bovine serum (FBS; PAN Biotech, Aidenbach, Germany) was injected into the peritoneal cavity. The resulting cell suspension was collected in a syringe, spun down and resuspended in RPMI 1640 medium with 10% FBS (PAN Biotech). Cells were stimulated ± 50 ng/ml endotoxin (lipopolysaccharide, serotype O55:B5; Sigma-Aldrich) for 2 h. Experimental set-up is summarized in Fig. S1C.

J774A.1 cells, a murine cell line showing a morphology of macrophages established from an adult, female mouse with reticulum cell sarcoma (DMSZ, Braunschweig, Germany), were cultured in Dulbecco's Modified Eagle Medium (PAN Biotech) supplemented with 10% FBS and 1% penicillin/streptomycin (PAN Biotech) at 37 °C in a humidified 5% CO_2_ atmosphere. At 80% confluency, cells were preincubated for 2 h with medium containing 50 μM JNK inhibitor (SP600125, Invivogen, Toulouse, France) and then treated with 10 ng/ml TNF-α (Sigma-Aldrich) for 2 h. The supernatant was collected at the end of the experiment. Experimental set-up is summarized in Fig. S1D. RNA from both experiments was isolated as detailed below.

### 2.3 Evaluation of liver damage and inflammation

Paraffin-embedded liver sections (4 μm) were stained with hematoxylin and eosin (Sigma-Aldrich) to evaluate liver histology using NAS as detailed by Kleiner et al. [23]. A commercially available naphthol AS-D chloroacetate esterase staining kit (Sigma-Aldrich) was used to detect neutrophil granulocytes as detailed previously [24]. Hepatic fibrosis and collagen depositions in liver sections were determined by staining with Picrosirius red and counterstained with fast green (Sigma-Aldrich, Steinheim, Germany) as detailed previously [25]. Representative pictures of all stainings were captured using a microscope integrated camera (LeicaDM4000 B LED, Leica, Wetzlar, Germany). Number of neutrophil granulocytes was counted per microscopic field in liver sections. For each tissue section the mean was determined from 8 fields (200× magnification). ALT activity in plasma was measured in a routine laboratory (Veterinary Medical University of Vienna, Vienna, Austria).

### 2.4 Griess assay

NO_2_^−^ levels in liver and cell culture supernatant were measured using the Griess reagent assay according to the manufacturer (Promega GmbH, Madison, WI, USA) and normalized to liver protein concentration.

### 2.5 ELISAs and MPO activity

Concentrations of total PAI-1, IL6 and IL1β protein were measured in liver tissue using commercially available ELISA-Kits (PAI-1: Innovative Research, Inc; Michigan, USA, IL6 and IL1β: DuoSet ELISA Kits, R&D Systems, Minneapolis, USA). MPO activity was measured in homogenized liver tissue as detailed before [26] and normalized to protein concentration.

### 2.6 Immunohistochemical staining

Sections of paraffin-embedded small intestinal tissue (4 μm) were stained for *anti*-zonula occludens 1 (*anti*-ZO-1, Invitrogen, USA) as previously described [27]. Representative pictures were taken using a microscope with an integrated camera (Leica DM6B & DMC4500, Leica, Germany).

### 2.7 Western blot

Cytosolic and nuclear proteins were isolated from liver tissue as detailed before [28]. Protein lysates and plasma were separated on 10% and 15% SDS-polyacrylamide gels, respectively, and transferred to polyvinylidene difluoride membranes (Bio-Rad Laboratories, Hercules, California, USA). Membranes were further incubated at 4 °C overnight with specific primary antibodies (adiponectin, histone, JNK, NFκB, phospho-JNK, phospho-NFκB, Cell Signaling Technology, Massachusetts, USA and β-actin, Santa Cruz Biotechnology, Dallas, TX, USA) and respective secondary antibodies (anti-rabbit or anti-mouse; Cell Signaling Technology, Massachusetts, USA). To detect protein bands, a commercially available kit was used (Super Signal Western Dura kit, Thermo Fisher Scientific, Waltham, MA, USA), and densitometric analysis were performed using ChemiDoc XRS System (Bio-Rad Laboratories, Hercules, CA, USA) as detailed previously [29].

### 2.8 RNA isolation, cDNA synthesis and real time PCR

Total RNA was extracted from liver tissue and cells and cDNA was synthetized using commercially available kits (PeqGold Trifast, VWR International GmbH, Vienna, Austria; Reverse Transcription System, Promega GmbH) as detailed elsewhere [24]. Real-time PCR were performed using primers listed in Table S1 and the expressions of the respective genes were normalized to 18S as previously described [9].

### 2.9 Measurement of bacterial endotoxin

Concentration of bacterial endotoxin in portal plasma was measured using a commercially available reporter gene assay (InvivoGen, Toulouse, France), which detects TLR4 ligands, as detailed previously [30].

### 2.10 Everted sac model of mice *ex vivo*

Small intestines (n = 4–6) from naïve male C57BL/6J mice and TNFα^−/−^ mice were collected and everted and tissue sacs were built as previously described in detail [21]. In brief, tissue sacs were filled with 1× Krebs-Henseleit-bicarbonate buffer supplemented with 0.2% bovine serum albumin (KRH buffer). After being equilibrated in gassed KRH buffer (95% O_2_/5% CO_2_) for 5 min at 37 °C, sacs were further incubated for 1 h at 37 °C in gassed KRH buffer ± 10 mM sucrose (Suc). After 55 min, 0.1% xylose was added to the incubation solutions for additional 5 min. Similarly, to assess intestinal permeability, everted tissue sacs from feeding experiments were incubated for 5 min in 0.1% xylose in KRH buffer. Concentration of xylose in collected liquids of everted gut sacs was measured using a modified protocol based on phloroglucinol as published before [31].

### 2.11 Statistics

The data are presented as means ± standard error of mean (SEM). Statistical analysis was performed using PRISM (version 7.03, GraphPad Software, Inc.). Grubb's test was used to determine outliers before further statistical analysis. Homogeneity of variances was tested and data were log-transformed if data were not normal distributed or in case of inhomogeneity of variances before performing further statistical tests. One- and two-factorial analysis of variance (ANOVA), respectively, were used to assess significant differences (*p* < 0.05).

## 3 Results

### 3.1 Markers of liver damage, inflammation, and fibrosis in C57BL/6J mice fed a sucrose-, fat-, and cholesterol-rich diet (SFC) and treated with an *anti*-TNFα antibody for 1 week

**Fig. 1:**
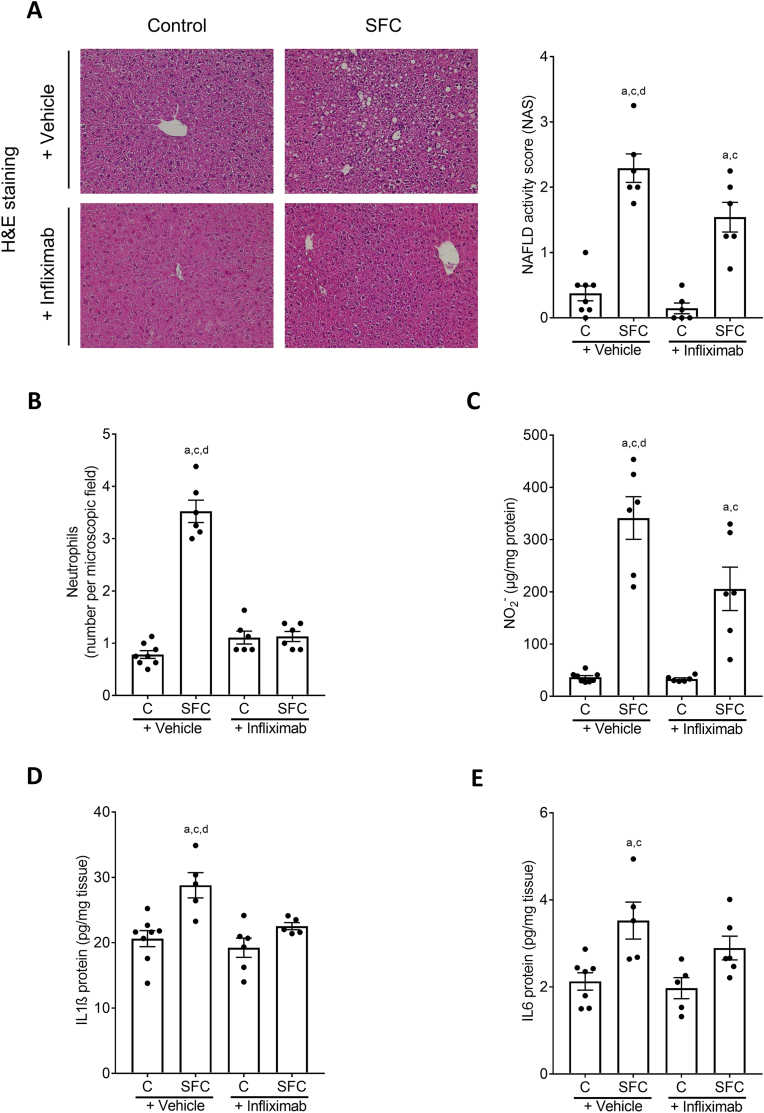
**Effect of infliximab on indices of liver damage and inflammatory markers in C57BL/6J mice fed a SFC for 8 weeks.** (A) Representative pictures (magnification 200×) of hematoxylin and eosin (H&E) staining in liver tissue and NAFLD activity score (NAS) in liver tissue, (B) number of neutrophil granulocytes per microscope field in liver tissue, (C) NO_2_^−^ concentration and protein concentration of (D) interleukin 1β (IL1β) as well as (E) interleukin 6 (IL6) in liver tissue. Data are shown as mean ± SEM, n = 6–8, except for (D) and (E): n = 5–8. ^a^*p* < 0.05 compared to C-fed + Vehicle-treated mice, ^c^*p* < 0.05 compared to C-fed + Infliximab-treated mice, ^d^*p* < 0.05 compared to SFC-fed + Infliximab-treated mice. C, control diet; SFC, sucrose-, fat-, and cholesterol-rich diet.

Markers of liver damage as determined by NAFLD activity score (NAS) and counting neutrophils were significantly lower in SFC-fed mice treated with infliximab compared to SFC-fed mice treated with vehicle (Fig. 1A and B, Table 1). The treatment with the *anti*-TNFα antibody had no effect on fat accumulation in liver tissue of SFC-fed mice whereas inflammation was dampened significantly (Table 1). Despite similar food intake, both absolute body weight and body weight gain were significantly higher in SFC-fed mice treated with vehicle than in controls, while no differences were found between SFC-fed mice treated with infliximab and all other groups. Absolute liver weight was also higher in SFC-fed mice treated with vehicle than in controls and did not differ between SFC-fed mice treated with infliximab and all other groups. Liver to body weight ratio and alanine aminotransferase (ALT) activity in plasma were similar between groups. Activity of myeloperoxidase (MPO) in liver tissue was also significantly higher in SFC-fed mice treated with vehicle compared to both C-fed groups, while MPO activity in livers of SFC-fed mice treated with infliximab did not differ from control diet (C)-fed mice or SFC-fed mice treated with vehicle (Table 1). Nitrite (NO_2_^−^) and interleukin 1β (IL1β) protein concentrations in liver were also higher in livers of SFC-fed mice treated with vehicle compared to all other groups. However, while being significantly lower than in the SFC-fed group, NO_2_^−^ levels in liver tissue of SFC-fed mice treated with infliximab were still significantly higher than in both control groups (Fig. 1C) whereas IL1β protein levels were similar to controls (Fig. 1D). Protein concentration of interleukin 6 (IL6) in livers of SFC-fed mice was significantly higher compared to the two C-fed groups but was not different from SFC-fed mice treated with infliximab. Also, IL6 protein concentration in livers of the latter did not differ from either other group (Fig. 1E). Furthermore, being in line with the findings that mice only displayed early signs of MASH, markers of fibrosis like mRNA expressions of *alpha smooth muscle actin* (*aSma*) and *collagen type I alpha1* (*Col1a1*) and Sirius red staining in liver tissue were similar between groups (Table 1). Representative pictures of the Sirius red staining are shown in Fig. S2A. As infliximab has been used successfully in the treatment of inflammatory bowel disease (IBD) [32] and the development of MASLD has been associated with impairments of intestinal barrier function [33], we next determined levels of endotoxin in portal plasma being indicative of intestinal barrier dysfunction. Bacterial endotoxin levels were significantly higher in both SFC-fed groups compared to C-fed mice (Table 1).

**Table 1:** Caloric intake, body and liver weight, liver damage and intestinal permeability of C57BL/6J mice fed a SFC concomitantly treated with infliximab or vehicle.

	Control	SFC	Control	SFC
	+ Vehicle		+ Infliximab	
**Caloric intake (kcal/g/bw)**	0.45 ± 0.00	0.43 ± 0.01	0.43 ± 0.01	0.44 ± 0.01
**Body weight (g)**	28.1 ± 0.5	31.4 ± 0.5^a,c^	28.5 ± 0.9	29.4 ± 0.6
**Absolute body weight gain (g)**	5.0 ± 0.7	8.7 ± 0.5^a,c^	4.7 ± 0.6	6.5 ± 0.9
**Liver weight (g)**	1.4 ± 0.0	1.7 ± 0.1^a,c^	1.4 ± 0.1	1.6 ± 0.1
**Liver:body weight ratio (%)**	4.9 ± 0.1	5.2 ± 0.1	5.0 ± 0.1	5.4 ± 0.2
**Steatosis (NAS)**	0.34 ± 0.1	1.83 ± 0.1^a,c^	0.15 ± 0.1	1.38 ± 0.2^a,c^
**Inflammation (NAS)**	0.00 ± 0.0	0.46 ± 0.1^a,c,d^	0.00 ± 0.0	0.17 ± 0.1^a,c^
**ALT (U/L)**	17.3 ± 2.2	27.2 ± 2.8	24.0 ± 5.3	28.3 ± 3.0
**MPO activity (% over control)**	100.0 ± 8.9	343.6 ± 97.1^a,c^	111.7 ± 17.3	153.3 ± 24.9
**Endotoxin (OD 655 nm)**	0.18 ± 0.01	0.27 ± 0.01^a,c^	0.17 ± 0.01	0.30 ± 0.02^a,c^
***aSma* mRNA expression**	100.0 ± 20.3	119.2 ± 26.1	98.8 ± 14.4	119.0 ± 28.3
***Col1a1* mRNA expression**	100.0 ± 14.7	151.9 ± 44.8	159.3 ± 25.3	165.4 ± 22.7

Data are shown as mean ± SEM, n = 6–8; ^a^*p* < 0.05 compared to C-fed + Vehicle-treated mice, ^c^*p* < 0.05 compared to C-fed + Infliximab-treated mice, ^d^*p* < 0.05 compared to SFC-fed + Infliximab-treated mice. aSma, alpha smooth muscle actin; ALT, alanine aminotransferase; Col1a1, collagen type I alpha1; C, control diet; MPO, myeloperoxidase; NAS, NAFLD activity score; SFC, sucrose-, fat-, and cholesterol-rich diet.

### 3.2 Markers of liver damage, fibrosis and glucose metabolism in wild-type and TNFα^−/−^ mice fed a SFC

**Fig. 2:**
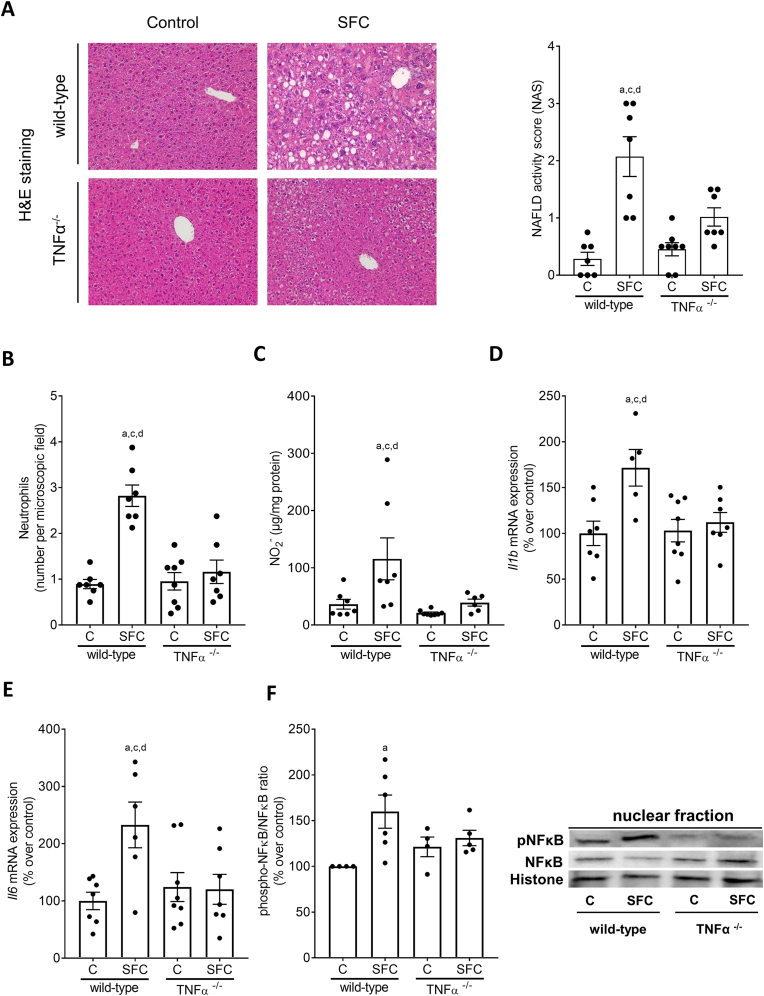
**Effect of a SFC fed for 9 weeks on indices of liver damage, inflammation, and pro-inflammatory markers as well as phosphorylation of NFκB in livers of wild-type and TNFα**^**−/−**^**mice.** (A) Representative pictures (magnification 200×) of hematoxylin and eosin (H&E) staining in liver tissue and NAFLD activity score (NAS) in liver tissue, (B) number of neutrophil granulocytes per microscope field in liver tissue, (C) hepatic NO_2_^−^ concentration, mRNA expression of (D) *interleukin 1b* (*Il1b*) and (E) *interleukin 6* (*Il6*) in liver tissue, (F) relative levels of phosphorylated NFκB protein in the nuclear fraction of the liver as well as representative pictures of the blots. Data are shown as mean ± SEM, n = 6–8, except for (F) n = 4–6. ^a^*p* < 0.05 compared to C-fed wild-type mice, ^c^*p* < 0.05 compared to C-fed TNFα^−/−^ mice, ^d^*p* < 0.05 compared to SFC-fed TNFα^−/−^ mice. C, control diet; SFC, sucrose-, fat-, and cholesterol-rich diet.

**Fig. 3:**
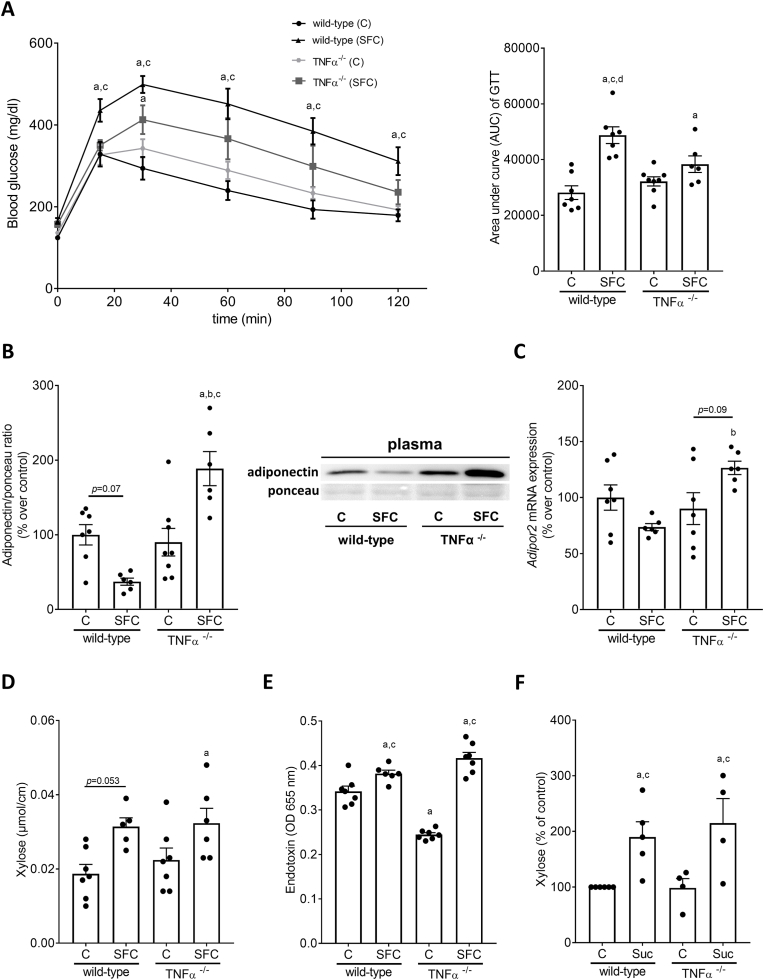
**Effect of a SFC fed for 9 weeks on glucose tolerance, adiponectin in plasma and *adiponectin receptor 2* (*Adipor2*) expression in liver tissue as well as markers of intestinal permeability in wild-type and TNFα**^**−/−**^**mice.** (A) Blood glucose levels during glucose tolerance test (GTT) and area under the curve (AUC) of blood glucose concentration, (B) adiponectin protein concentration in plasma and representative pictures of the blots, (C) mRNA expression of *Adipor2* in liver tissue of wild-type C57BL/6J mice and TNFα^−/−^ mice. (D) Xylose permeation in small intestine tissue and (E) endotoxin levels in plasma of wild-type C57BL/6J mice and TNFα^−/−^ mice. (F) Xylose permeation in everted gut sacs of naïve wild-type C57BL/6J mice and TNFα^−/−^ mice challenged with 10 mM sucrose (Suc). Data are shown as mean ± SEM, n = 6–8, except for (C) and (E): n = 6–7, for (D): n = 5–7 and for (F): n = 4–6. ^a^*p* < 0.05 compared to C-fed wild-type mice, ^c^*p* < 0.05 compared to C-fed TNFα^−/−^ mice, ^d^*p* < 0.05 compared to SFC-fed TNFα^−/−^ mice. C, control diet; SFC, sucrose-, fat-, and cholesterol-rich diet.

To further determine the role of TNFα in the development of early MASH in a non-obese setting, TNFα^−/−^ mice and wild-type mice were pair-fed the SFC or C diet for 9 weeks. Despite similar caloric intake and not being overweight, only SFC-fed wild-type mice developed obvious signs of MASLD (Table 2). Indeed, total NAS and numbers of neutrophils in liver tissue were significantly higher in SFC-fed wild-type mice compared to both C-fed groups and SFC-fed TNFα^−/−^ mice. No differences were found when comparing control groups with SFC-fed TNFα^−/−^ mice (Fig. 2A and B). However, when comparing steatosis scores between groups, scores for SFC-fed TNFα^−/−^ mice were significantly higher than those of controls, while scores for inflammation were alike between control groups and SFC-fed TNFα^−/−^ mice (Table 2). Absolute liver weight, liver to body weight ratio and ALT activity in plasma were similar between the two SFC-fed groups being significantly higher than in both C-fed groups (Table 2). In addition, markers of hepatic fibrosis were determined. Neither mRNA expression of *aSma* and *Col1a1* in livers of mice (Table 2) nor staining of Sirius red revealed any differences between groups (Fig. S2B). In contrast, concentrations of NO_2_^−^ as well as *Il1b, Il6,* and *monocyte chemoattractant protein 1 (Mcp1)* mRNA expression were significantly higher in livers of SFC-fed wild-type mice than in all other groups. In contrast, concentrations were alike between SFC-fed TNFα^−/−^ and control groups (Fig. 2C–E, Table 3). Protein levels of the acute phase protein plasminogen activator inhibitor 1 (PAI-1) were also only significantly higher in livers of SFC-fed wild-type mice compared to all other groups while not differing between C-fed groups and SFC-fed TNFα^−/−^ mice (Table 3). Phosphorylated NFκB in nuclear extracts was at the level of controls in livers of TNFα^−/−^ mice whereas being significantly higher in nuclear extracts in livers of SFC-fed wild-type mice (Fig. 2F). Fasting glucose blood levels were significantly higher in both SFC-fed groups compared to their respective controls (Table 2). In contrast, area under the curve (AUC) of the glucose tolerance test (GTT) was significantly higher in SFC-fed wild-type mice than in all other groups. While AUC of the GTT was significantly lower in TNFα^−/−^ fed SFC than in wild-type mice, AUC was still significantly higher in SFC-fed TNFα^−/−^ mice than in C-fed wild-type mice (Fig. 3A). Somewhat in line with these findings, *insulin receptor substrate 1* (*Irs1*) mRNA expression in liver tissue was also significantly higher in SFC-fed wild-type mice compared to all other groups whereas *Irs2* mRNA expression was significantly higher in SFC-fed wild-type mice compared to controls and by trend compared to SFC-fed TNFα^−/−^ mice (*p* = 0.07, Table 3). No differences were found in *Irs1* and *Irs2* mRNA expression in liver between C-fed groups and SFC-fed TNFα^−/−^ mice. Expression of *insulin receptor* (*Ir*) mRNA in liver was similar between groups (Table 3).

**Table 2:** Effect of a SFC fed for 9 weeks on caloric intake, body and liver weight as well as markers of liver damage, inflammation and glucose metabolism in wild-type and TNFα^−/−^ mice.

	wild-type mice		TNFα^−/−^ mice	
	Control	SFC	Control	SFC
**Caloric intake (kcal/g/bw)**	0.39 ± 0.00	0.47 ± 0.01^a,c^	0.38 ± 0.00	0.46 ± 0.01^a,c^
**Body weight (g)**	27.7 ± 1.0	29.2 ± 0.7	27.3 ± 0.3	28.1 ± 0.5
**Absolute body weight gain (g)**	3.2 ± 0.7	6.0 ± 0.5^a^	4.2 ± 0.2	5.5 ± 0.2^a^
**Liver weight (g)**	1.2 ± 0.1	1.7 ± 0.1^a,c^	1.3 ± 0.0	1.6 ± 0.0^a,c^
**Liver:body weight ratio (%)**	4.5 ± 0.1	5.9 ± 0.2^a,c^	4.6 ± 0.0	5.8 ± 0.1^a,c^
**Steatosis (NAS)**	0.11 ± 0.0	1.20 ± 0.2^a,c,d^	0.25 ± 0.1	0.50 ± 0.1^a^
**Inflammation (NAS)**	0.21 ± 0.1	0.88 ± 0.2^d^	0.22 ± 0.1	0.45 ± 0.1
**ALT (U/L)**	13.2 ± 1.4	20.6 ± 1.5^a,c^	15.7 ± 0.7	20.3 ± 1.1^a,c^
**Fasting blood glucose (mg/dl)**	124.4 ± 5.4	165.3 ± 7.2^a,c^	136.3 ± 5.5	158.4 ± 5.9^a^
***aSma* mRNA expression**	100.0 ± 29.2	97.7 ± 27.5	123.2 ± 25.7	132.2 ± 20.6
***Col1a1* mRNA expression**	100.0 ± 8.9	229.9 ± 70.8	143.7 ± 17.2	133.8 ± 16.2

Data are shown as mean ± SEM, n = 6–8; ^a^*p* < 0.05 compared to C-fed wild-type mice, ^c^*p* < 0.05 compared to C-fed TNFα^−/−^ mice, ^d^*p* < 0.05 compared to SFC-fed TNFα^−/−^ mice. aSma, alpha smooth muscle actin; ALT, alanine aminotransferase; Col1a1, collagen type I alpha1; C, control diet; NAS, NAFLD activity score; SFC, sucrose-, fat-, and cholesterol-rich diet.

**Table 3:** Effect of a SFC fed for 9 weeks on hepatic markers of glucose metabolism and inflammation in wild-type and TNFα^−/−^ mice.

	wild-type mice		TNFα^−/−^ mice	
	Control	SFC	Control	SFC
***Mcp1* mRNA expression**	100.0 ± 13.5	405.3 ± 144.0^a,c,d^	81.0 ± 15.4	95.8 ± 13.2
**PAI-1 (ng/mg protein)**	0.016 ± 0.002	0.030 ± 0.004^a,c,d^	0.019 ± 0.001	0.018 ± 0.001
***Irs1* mRNA expression**	100.0 ± 10.3	242.4 ± 37.0^a,c,d^	132.2 ± 20.4	146.0 ± 16.0
***Irs2* mRNA expression**	100.0 ± 9.3	248.3 ± 37.6^a,c^	139.0 ± 26.1	136.5 ± 10.7
***Ir* mRNA expression**	100.0 ± 8.8	136.4 ± 12.5	115.4 ± 20.5	129.1 ± 10.1

Data are shown as mean ± SEM, n = 6–8; ^a^*p* < 0.05 compared to C-fed wild-type mice, ^c^*p* < 0.05 compared to C-fed TNFα^−/−^ mice, ^d^*p* < 0.05 compared to SFC-fed TNFα^−/−^ mice. C, control diet; SFC, sucrose-, fat-, and cholesterol-rich diet; Irs, insulin receptor substrate; Ir, insulin receptor; Mcp1, monocyte chemoattractant protein-1; PAI-1, plasminogen activator inhibitor 1.

### 3.3 Adiponectin in plasma and expression of adiponectin receptors in liver tissue in wild-type and TNFα^−/−^ mice fed a SFC

Protein levels of adiponectin suggested to be the counterplayer of TNFα [34] were by trend lower in plasma of SFC-fed wild-type mice compared to wild-type controls (*p* = 0.07). Adiponectin protein levels in plasma of SFC-fed TNFα^−/−^ mice were significantly higher than in all other groups (Fig. 3B). Moreover, expression of *adiponectin receptor 2 (Adipor2)* mRNA was significantly lower in livers of SFC-fed wild-type than in SFC- fed TNFα^-/-^ mice and by trend lower in livers of C-fed TNFα^−/−^ compared to SFC-fed TNFα^-/-^ mice (SFC-fed wild-type vs. SFC-fed TNFα^−/−^ *p* < 0.05; SFC-fed TNFα^-/-^ vs. C-fed TNFα^−/−^ *p* = 0.09) (Fig. 3C). Expression of *Adipor1* mRNA was not detectable.

### 3.4 Markers of intestinal permeability in wild-type and TNFα^−/−^ mice fed a SFC

Permeability as assessed by xylose permeation in small intestinal tissue sacs *ex vivo* at time of killing, was significantly and by trend (*p* = 0.053) higher in both SFC-fed groups compared to wild-type controls (Fig. 3D). Also, bacterial endotoxin levels in portal plasma shown to be a mediator of the induction of TNFα expression but also NFκB activation [35] were significantly higher in both SFC-fed groups compared to the two C-fed groups (Fig. 3E). Interestingly, in C-fed TNFα^−/−^ mice, concentration of endotoxin was even lower than in C-fed wild-type mice (Fig. 3E). Moreover, concentrations of ZO-1 protein were lower in SFC-fed wild-type and TNFα^−/−^ mice compared to C-fed mice (representative pictures see Fig. S3). Permeation of xylose was similarly significantly increased in everted small intestinal tissue sacs of naïve wild-type and TNFα^−/−^ mice challenged with physiological doses of sucrose (10 mM) when compared to sacs only challenged with Krebs-Henseleit-bicarbonate buffer supplemented with 0.2% bovine serum albumin (KRH buffer) (Fig. 3F).

### 3.5 Effect of endotoxin on cytokine release in peritoneal cavity cells (PCCs) isolated from wild-type and TNFα^−/−^ mice

**Fig. 4:**
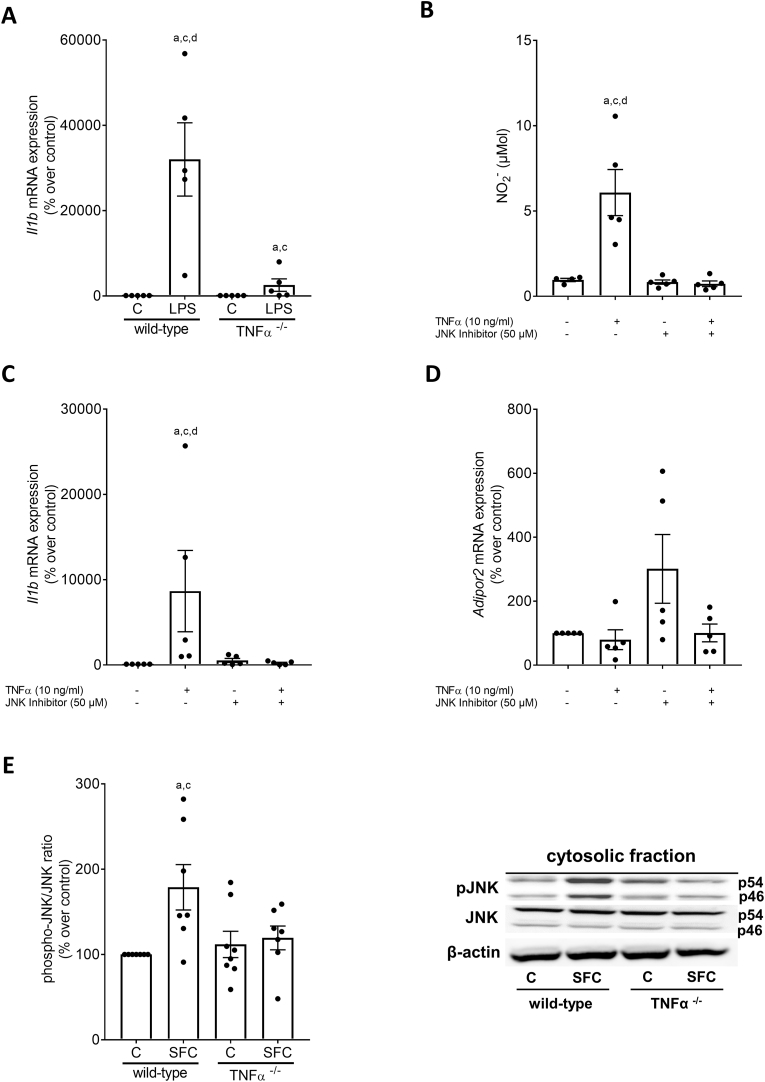
**Pro-inflammatory markers in peritoneal cavity cells (PCCs) isolated from wild-type and TNFα**^**−/−**^**mice challenged with LPS as well as J774A.1 cells challenged with TNFα in the presence of a JNK inhibitor (SP600125) and phosphorylation of JNK in livers of SFC-fed wild-type and TNFα−/− mice.** (A) Expression of *interleukin 1b* (*Il1b)* mRNA of PCCs stimulated with LPS (50 ng/ml) for 2h, (B) NO_2_^−^ concentration in supernatant, mRNA expression of (C) *interleukin 1b* (*Il1b) and (D) adiponectin receptor 2 (Adipor2)* of J774A.1 cells preincubated with an JNK Inhibitor (50 μM) for 2h and stimulated with TNFα (10 ng/ml) also for 2h. (E) Relative phospho-JNK protein concentration and representative pictures of blots in SFC-and C-fed wild-type and TNFα^−/−^ mice. Data are shown as mean ± SEM, n = 5, except for (B) n = 4–5 and for (E) n = 6–8. For (A): ^a^*p* < 0.05 compared to untreated PCCs of wild-type mice, ^c^*p* < 0.05 compared to untreated PCCs of TNFα^−/−^ mice, ^d^*p* < 0.05 compared to LPS-treated PCCs of TNFα^−/−^ mice. For (B)–(D): ^a^*p* < 0.05 compared to untreated J774A.1 cells, ^c^*p* < 0.05 compared to J774A.1 cells preincubated with a JNK inhibitor, ^d^*p* < 0.05 compared to J774A.1 cells incubated with TNFα and a JNK inhibitor. For (E): ^a^*p* < 0.05 compared to C-fed wild-type mice, ^c^*p* < 0.05 compared to C-fed TNFα^−/−^ mice. C, control; SFC, sucrose-, fat-, and cholesterol-rich diet; LPS; endotoxin-stimulated cells.

In endotoxin-treated PCCs isolated from wild-type mice *Il1b* mRNA expression was significantly higher than in naïve cells (+∼300-fold). In contrast, while still being significantly higher than in unstimulated cells, *Il1b* mRNA expression in PCCs isolated from TNFα^−/−^ mice was only ∼25-fold higher than in unstimulated controls (Fig. 4A).

### 3.6 Effect of TNFα and a JNK inhibitor on cytokine release and *Adipor2* expression in J744A.1 cells as well as JNK phosphorylation in wild-type and TNFα^−/−^ mice fed a SFC

To further determine through which signaling cascade TNFα may induce expression of pro-inflammatory cytokines in immune cells e.g., Kupffer cells, J744A.1 cells were employed as model of Kupffer cells and were stimulated with TNFα for 2 h in the presence or absence of the JNK inhibitor SP600125. The increase in NO_2_^−^ concentration in cell supernatant and the induction of *Il1b* mRNA expression found in cells stimulated with TNFα were significantly blocked in cells pre-treated with the JNK inhibitor (Fig. 4B and C). Indeed, while expression of *Adipor2* mRNA was not altered (Fig. 4D), both NO_2_^−^ concentration and *Il1b* mRNA expression were at the level of controls 2 h after being exposed to TNFα (Fig. 4B and C). In line with these findings, concentration of phosphorylated JNK in liver tissue was also significantly higher in livers of SFC-fed wild-type mice compared to controls while similar differences were not found when comparing phosphorylated JNK in liver tissue of SFC-fed TNFα^−/−^ mice with their respective controls (Fig. 4E).

## 4 Discussion

Among many inflammatory markers, TNFα has emerged as one of the key cytokines influencing intermediary metabolism and subsequently the development of metabolic diseases including MASLD (for overview see [36]). Indeed, results of animal and human intervention studies suggest that an attenuation of the development of MASLD and/or improvement of the disease is often afflicted with a lowering of *Tnfa* mRNA expression in liver tissue or circulating TNFα protein levels in blood [37,38]. However, molecular mechanisms underlying the devastating effects of TNFα in the development of MASLD have not yet been clarified. In the present study, the loss of TNFα, either through an inhibition with a specific antibody or a genetic deletion, was associated with a marked protection from inflammatory alterations in liver tissue. These findings are in line with our own previous findings and those of others employing either TNFα antibodies or TNFα receptor knockout mice [[17], [18], [19], [20]]. As mice showed only early signs of MASLD, ALT activities in plasma were only slightly higher in SFC-fed mice and no signs of fibrosis were detected. The latter findings are in line with previous findings of us and others suggesting that an 8–9 week long feeding of a sugar-, fat- and cholesterol-rich diet is not sufficient to induce marked signs of inflammation [39,40]. Also, as hepatic fat accumulation was almost similar between SFC-fed groups, ALT activities were also similarly elevated in both SFC-fed groups. Indeed, it has been shown before that ALT activity in plasma may reflect hepatic fat accumulation more closely than inflammation [41]. Hepatic fat accumulation was not affected by the one-week treatment with the *anti*-TNFα antibody infliximab. These results contrast findings of others reporting a reversion of steatosis in rats fed a high-fat diet when being treated with infliximab [20]. The lack of efficacy of the *anti*-TNFα antibody to reduce hepatic fat accumulation in the present study might have been related to the duration of the treatment (1 week vs. 10 and more days in other studies) but also the dose used (every other day 10 mg/kg bw i.p. vs. twice daily 100 μg in 100 μl saline/dose i.p.) [20]. Still, it remains to be determined if, when given over an extend period or in settings of more progressed MASLD and in humans, infliximab may also reduce hepatic fat accumulation.

Also in line with the findings of others [42], mRNA expression of *Adipor2* in liver tissue and adiponectin protein levels in plasma were lower in SFC-fed than in C-fed wild-type mice while expression of *Adipor2* in liver and protein levels of adiponectin in plasma were higher in SFC-fed TNFα^−/−^ mice than in SFC-fed wild-type mice. It has been proposed before in studies with overweight high-fat diet-fed mice that TNFα is involved in the regulation of adiponectin secretion and multimerization in adipose tissue and that this regulation may be related to an activation von JNK, ERK, and p38 [43]. If mechanisms alike are involved in the alterations found in the present study remains to be determined.

In line with the findings for pro-inflammatory cytokines but also adiponectin, SFC-fed TNFα^−/−^ mice were also markedly protected from the development of glucose intolerance and impairments of insulin signaling in liver tissue e.g., alterations of expression of *Irs1* and *Irs2*. It has been shown before that targeting TNFα through antibodies or knocking out its receptors glucose tolerance can be improved in rodent models [44,45]. Also, an induction of IRS1 has been shown by others, too, to be associated with the development of insulin resistance in diet-induced MASLD [46]. Somewhat contrasting our findings but also some of the previous studies reporting that a loss of TNFα may improve glucose tolerance in settings of MASLD and diabetes type 2, it has been shown by Wu et al. that a downregulation of TNFα in TNFR1/TNFR2 double knockouts employing an Adenovirus-associated virus-shRNA TNFα worsened glucose homeostasis [44], further suggesting that TNFα may be pleiotropic. Indeed, in the present study, fasting glucose levels of SFC-fed TNFα^−/−^ mice were similar elevated as those of wild-type mice. Also, it has been shown that an acute administration of TNFα lowers basal plasma insulin levels through yet not fully understood mechanism in healthy young men [47].

Taken together our results suggest that an inhibition or genetic deletion of TNFα in settings of diet-induced non-obese/lean MASLD may diminish the development of MASLD and insulin resistance in mice and that this is associated with a protection from the induction of pro-inflammatory cytokines, and alterations in adiponectin signaling. However, these data do not preclude that in later stages of the disease, a loss of TNFα may also have adverse effects. Rather our data suggest that in early stages of the disease targeting TNFα and related signaling cascades may have beneficial effects, even when dietary habits remain unchanged. Still further studies are needed to determine if similar effects are also found in non-obese/lean patients with MASLD.

### 4.1 The loss of TNFα does not affect intestinal barrier function in small intestine and translocation of bacterial endotoxin

Results of studies employing animal models and patients with MASLD suggest that alterations of intestinal barrier function and an increased translocation of bacterial endotoxin but also other PAMPs are critical in the development of MASLD and the induction of hepatic TNFα expression (for overview see [48]). Indeed, results obtained in experimental models of MASLD suggest that targeting the endotoxin-TLR4 signaling cascade not only attenuated the development of MASLD but also the expression of TNFα [49]. Also, in models of IBD it has been shown that TNFα itself may add to the development of intestinal barrier dysfunction [50]. It also has been shown that *anti*-TNFα treatments may reduce mucosal inflammation and restore intestinal permeability in IBD patients and may alter intestinal microbiota composition [51]. Moreover, in the study of Manka et al. it was shown that the protective effects of the *anti*-TNFα treatment were associated with an improvement of hepatic steatosis in patients with Crohn's Disease [52]. Somewhat in contrast, in the present study, bacterial endotoxin levels suggested to be indicative of intestinal barrier dysfunction [53] were significantly higher in SFC-fed mice regardless of strain or treatment when compared with the respective controls. Supporting these findings, xylose permeation in small intestine was also similar between SFC-fed mice, regardless of strain. Also, when challenging small intestinal tissue of naïve wild-type and TNFα^−/−^ mice with sucrose, xylose permeation was also similarly elevated in both groups compared to controls. These data suggest that in settings of diet (sucrose)-induced non-obese MASLD, TNFα may not be a critical factor in the development of intestinal barrier dysfunction. Indeed, some more recent findings of our group suggest that nutritional compounds like fructose being also present in sucrose may alter intestinal barrier function through more direct mechanisms [21,54]. However, it remains to be determined if intestinal barrier dysfunction associated with later stages of the disease e.g., MASH and fibrosis is also unrelated to TNFα and if this is also true for humans with MASLD. Also, if intestinal microbiota composition is altered by the treatment with infliximab or the genetic deletion of TNFα in settings of diet-induced MASLD and how this impacts disease development also remains to be determined.

### 4.2 TNFα regulated the expression of pro-inflammatory markers like IL1β and NO_2_^−^ in immune cells through JNK-dependent signaling cascades

Results of several studies have shown that bacterial endotoxin can lead to an induction of TNFα but also NO_2_^−^ and cytokines like IL1β and IL6 in macrophages and hepatic Kupffer cells through TLR4 and NFκB-dependent signaling cascades [55,56]. Also, it has been shown before that TNFα is a counterplayer of adiponectin and that in Kupffer cells a treatment with adiponectin attenuated the endotoxin-dependent induction of TNFα [57]. In the present study, the blockage of TNFα with an antibody and the genetic deletion of the cytokine were both associated with an attenuation of the induction of NO_2_^−^ levels as well as *Il1b* and *Il6* mRNA expression in liver tissue of SFC-fed mice. Also, the phosphorylation of NFκB was almost at the level of controls in livers of TNFα^−/−^ mice. It has been proposed before that TNFα may be a key mediatory of the subsequent effects of bacterial endotoxin related to the activation of NFκB and the resulting induction of pro-inflammatory mediators like IL1β, IL6 and NO_2_^−^ [58,59]. Indeed, it has been shown that upon exposure to endotoxin, Kupffer cells release TNFα within 2 h in a dose-dependent manner [60] whereas an induction of the release of IL1β, IL6, and also NO_2_^−^ was detected after 6–24 h [60,61]. Results of animal studies mimicking liver diseases of various etiologies have suggested that targeting TNFα release with specific antibodies is associated with decreased expressions of several cytokines including IL6 [62]. In support of the hypothesis that bacterial endotoxin may at least in part trigger the induction of other cytokines through TNFα-dependent signaling cascades, in endotoxin-challenged PCCs isolated from TNFα^−/−^ mice the induction of *Il1b* mRNA expression was dampened by ∼90% compared to PCCs isolated from wild-type mice (untreated wild-type PCCs vs. endotoxin-treated wild-type PCCs: +∼300-fold). It has been proposed that the TNFα-dependent regulation of other cytokines and adiponectin as well as its receptor 2 may involve an activation of JNK and dependent signaling cascades [43]. In the present study, the JNK inhibitor SP600125 attenuated the TNFα-induced increase of NO_2_^−^ in cell culture medium of J774A.1 cells and the induction of *Il1b* mRNA expression almost completely. In contrast, while we found that TNFα induced a marked down-regulation in expression of *Adipor2*, the JNK inhibitor only partly attenuated this effect of TNFα further suggesting that other signaling cascades may be critical in the TNFα-dependent regulation of *Adipor2* expression (e.g., PPARα) [34,43]. In line with the findings in cell culture experiments employing the JNK inhibitor, phosphorylation of JNK in livers of SFC-fed TNFα^−/−^ mice was also almost similar to that of controls. Taken together, our results add further weight to the hypothesis that an increased translocation of bacterial endotoxin may through TNFα- and JNK-dependent signaling cascades enhance the formation of cytokines like IL1β and IL6 and of pro-oxidative mediators like NO_2_^−^ thereby adding to the onset and probably also progression of MASLD. However, these results by no means preclude that bacterial endotoxin may also activate IL1β and IL6 as well as iNOS through TNFα-independent pathways; rather, our results suggest that in the early phase of MASLD, TNFα may be one of the key mediators in the development of diet-induced non-obese MASLD. Still further studies are at need to determine the role of TNFα in more detail in humans.

## 5 Conclusion

Taken together, results of the present study further bolster the hypothesis that TNFα is a key trigger in the onset of diet-induced non-obese MASLD and insulin resistance. Results of the present study also suggest that in mice in settings of diet-induced non-obese MASLD, TNFα is not a regulator of intestinal permeability but rather seems to trigger the induction and release of other inflammatory markers such as IL1β, IL6 and NO_2_^−^ through JNK-dependent signaling cascades in the liver. Furthermore, targeting TNFα e.g., with a specific antibody, seems at least in mice with non-obese MASLD to revert some of the early inflammatory alterations associated with the progression of the disease even in the absence of a change in diet. It has been suggested that TNFα may also promote liver regeneration and that blocking TNFα may have adverse effects and may even exacerbate the disease [63], whereas our data suggest that at least in lean mice with MASLD a short-term diminishment of TNFα may have beneficial effects on the liver. Further studies are needed to determine if targeting TNFα is also affected with a diminishment of inflammatory alterations in non-obese, lean patients with MASLD if the patients continue their regular diet and if these effects are also found in more progressed stages of the disease.

## Author contributions

Conceptualization, IB; data curation or formal analysis, KB, FJ, ABa, ABr, VS, RS, IB; funding acquisition, IB; investigation, KB, FJ, ABa, ABr, VS, RS, IB; supervision, IB; visualization, KB, IB; writing original draft preparation, KB and IB; writing – review and editing, KB, ABa and IB. All authors have read and agreed to the final version of the manuscript.

## Funding

This research received funding from the Austrian Science Fund (FWF, P 32164 (IB)).

## Declaration of competing interest

All authors declare no conflict of interest.

## Data Availability

Data will be made available on request.
