# Supplementary material for: TNFα is a key trigger of inflammation in diet-induced non-obese MASLD in mice
Source: Redox Biol. 2023 Sep 1;66:102870. doi: 10.1016/j.redox.2023.102870 (PMC10493600; doi:10.1016/j.redox.2023.102870)
Supplement: Multimedia component 1 [file mmc1.pdf]

Table S1: Primer sequences used for real-time PCR.

	Forward (5´- 3´)	*Reverse (5´-3´)*
18S	GTA ACC CGT TGA ACC CCA TT	CCA TCC AAT CGG TAG TAG CG
aSma	CTG ACA GAG GCA CCA CTG AA	CAT CTC CAG AGT CCA GCA CA
Adipor2	TGC CCT AGG TAG GCC CAA CA	TCC CAA AGT GCA CGA CCA GA
Col1a1	ACG TGG AAA CCC GAG GTA TG	CTT GGG TCC CTC GAC TCC TA
Il1b	GTC CGA CAG CAC AGA GGC TTT	TGG CTG TGG AGA AGC TGT GG
Il6	CCA CGC CTT CCC TAC TTC A	TGC AAG TGC ATC ATC GTT GTTC
Ir	CAT CCC GAA AGC GAA GAT CC	GAG TCC TGA TTG CAT GCC TGC AG
Irs1	GCT CTA GTG CTT CCG TGT CC	GTT GCC ACC CCT AGA CAA AA
Irs2	GAA GCG GCT AAG TCT CAT GG	GAC GGT GGT GGT AGA GGA AA
Mcp1	GTC CCT GTC ATG CTT CTG GG	GGC GTT AAC TGC ATC TGG CT

Adipor, adiponectin receptor 2; aSma, alpha smooth muscle actin; Col1a1, collagen type I alpha1; Il1b, interleukin 1 beta; Il6, interleukin 6; Ir, insulin receptor; Irs, insulin receptor substrate; Mcp1, monocyte chemoattractant protein-1.

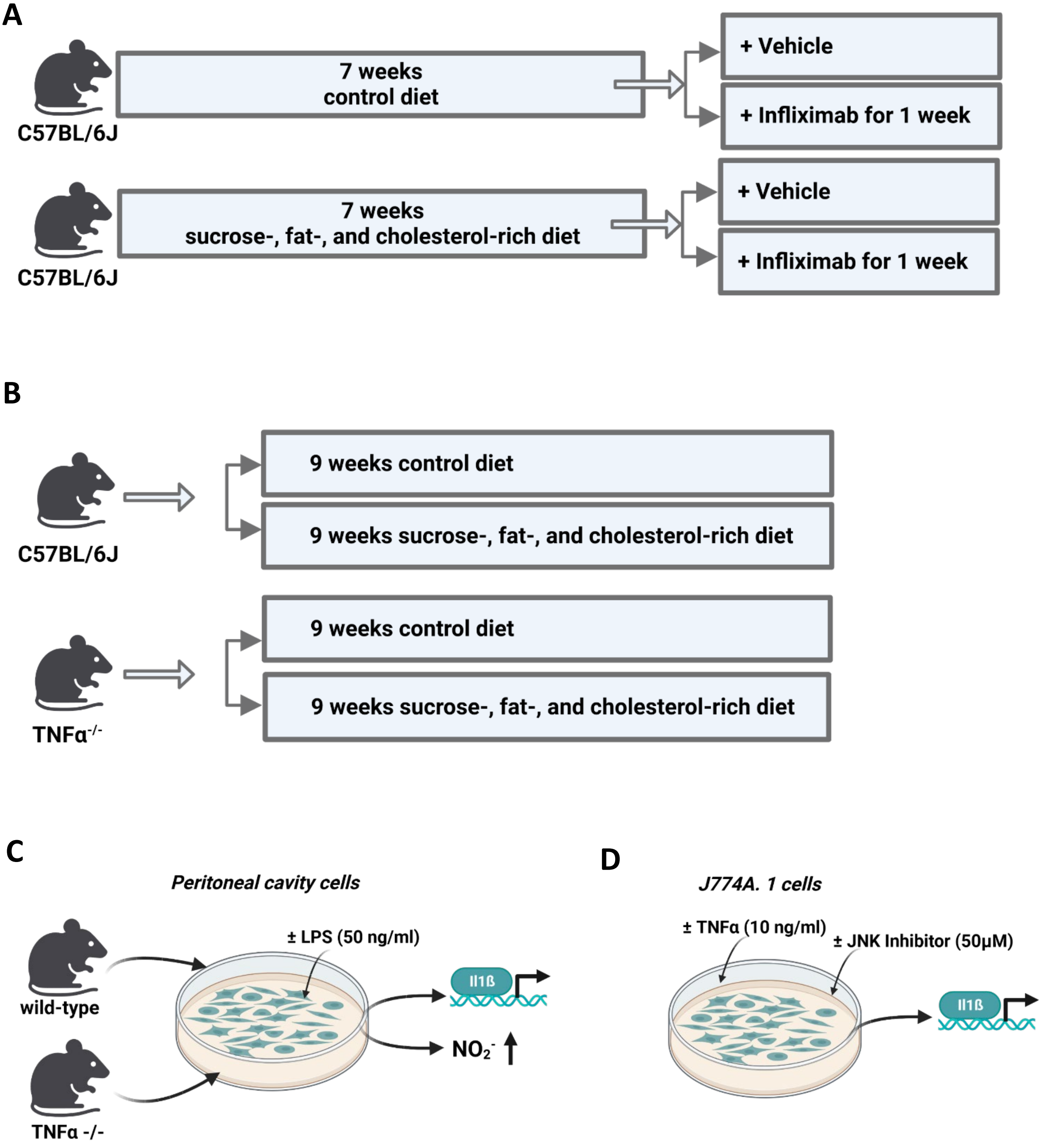

Figure S1: Schematic drawing of experimental set-ups. Experimental set-up of (A) intervention trial 1 and (B) intervention trial 2 of the *in vivo* experiment. Experiments in (C) peritoneal cavity cells (PCCs) isolated from naïve wild-type and TNFα$^{-/-}$ mice and (D) J774A.1 cells pre-treated with a JNK Inhibitor SP600125 (50 µM) for 2 hours and stimulated with TNFα (10 ng/ml) also for 2 hours.

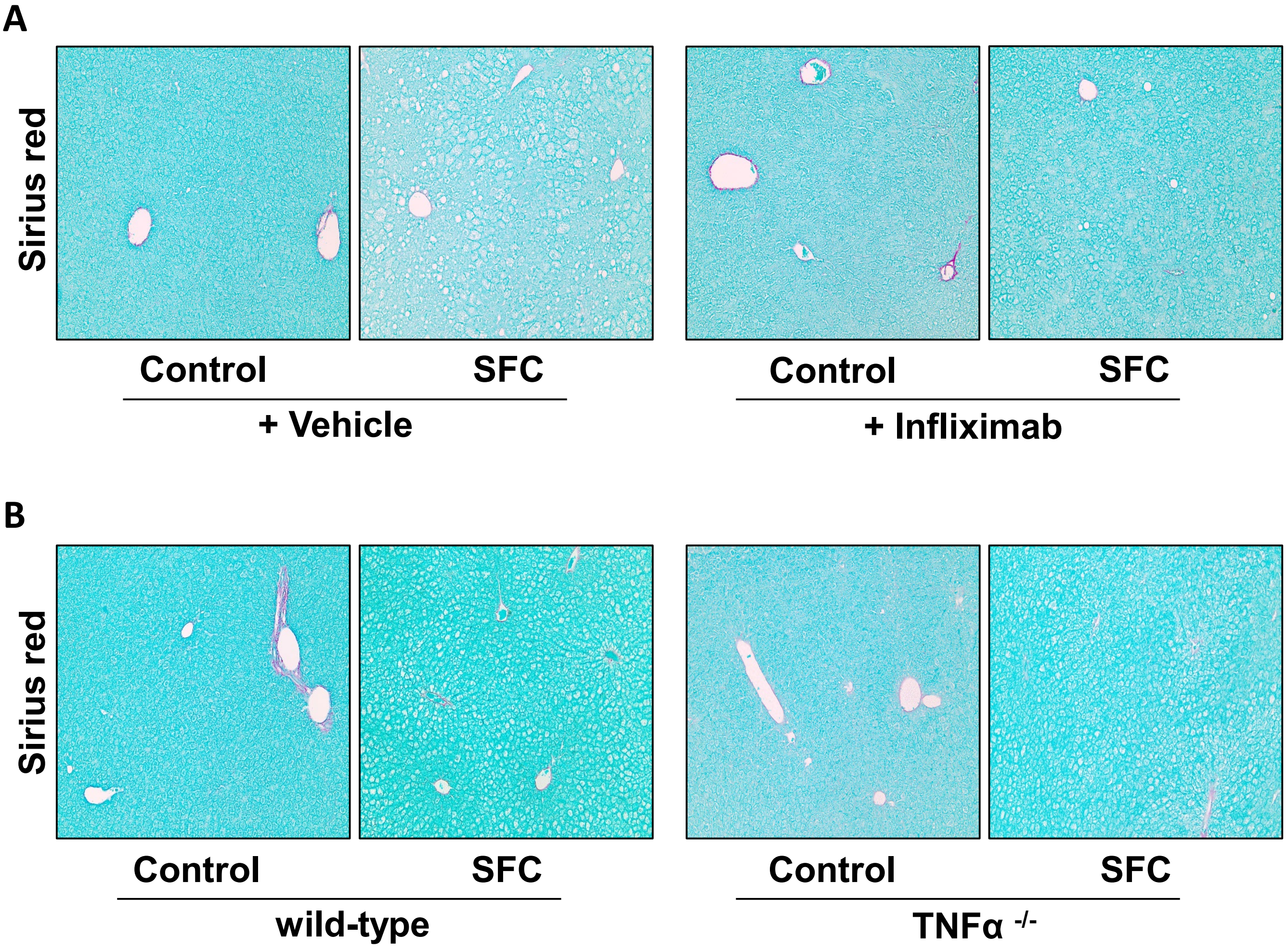

Figure S2: Representative pictures of Sirius red staining in liver tissue of SFC-fed (A) C57BL/6J mice treated with vehicle or Infliximab as well as (B) wild-type and TNFα$^{-/-}$ mice (magnification 200 x). C, control diet; SFC, sucrose-, fat-, and cholesterol-rich diet.

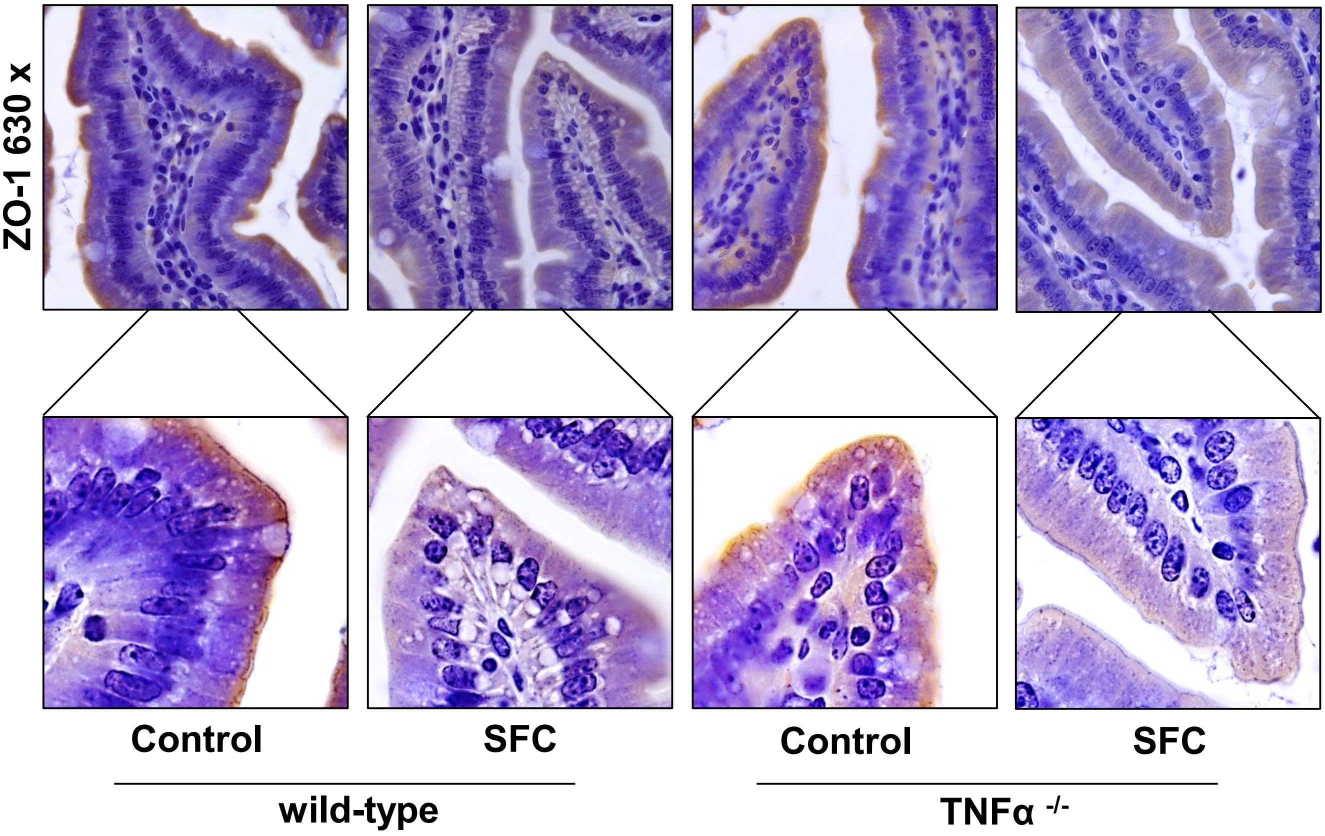

Figure S3: Effect of a SFC fed for 9 weeks on protein concentration of zonula occludens 1 (ZO-1) in small intestine of wild-type and TNFα$^{-/-}$ mice. Representative pictures of staining (magnification 630 x) and enlargement of villus tips. C, control diet; SFC, sucrose-, fat-, and cholesterol-rich diet.
